# Relationship between Methods of Loading and Unloading, Carcass Bruising, and Animal Welfare in the Transportation of Extensively Reared Beef Cattle

**DOI:** 10.3390/ani8070119

**Published:** 2018-07-17

**Authors:** Stella Maris Huertas, Rick E. A. M. Kempener, Frank J. C. M. van Eerdenburg

**Affiliations:** 1 Instituto de Biociencias Veterinarias, Facultad de Veterinaria, Universidad de la República, Lasplaces 1550, Montevideo PC 11600, Uruguay; stellamaris32@gmail.com; 2 Department of Farm Animal Health, Faculty of Veterinary Medicine, University of Utrecht, Yalelaan 7, 3584 CL Utrecht, The Netherlands; r.e.a.m.kempener@gmail.com

**Keywords:** carcass bruises, cattle transport, animal welfare, extensive production system

## Abstract

**Simple Summary:**

In Uruguay, extensive, welfare-friendly beef production is a substantial part of the economy and culture. Transport of beef cattle to the slaughterhouse compromises animal welfare. The objective of this study was to assess transport conditions related to carcass bruising. A total of 242 trucks with 8132 animals were assessed on loading, transport, unloading conditions, and carcass bruising. In 39.3% of the loadings only a flag was used. The fastest unloading time was performed using a flag only. Carcass bruises were assessed by trained observers inside the plant. Although the number of bruises was high, there were no grade 3 bruises, the deepest and severe ones. It appeared that animal welfare training of truck drivers worked out well and the use of flags to move animals increased compared to a previous study in 2008.

**Abstract:**

In Uruguay, extensive, welfare-friendly beef production is a substantial part of the economy and culture. Transport of beef cattle to slaughterhouse compromises animal welfare. The objective of this study was to assess transport conditions related to carcass bruising. A total of 242 trucks with 8132 animals were assessed on loading, transport, unloading conditions, and carcass bruising. Average loading time was 26 min and 21 s and the perception of the truck drivers was correlated with the time took for loading and the use of devices. In 39.3% of the loadings only a flag was used. The average unloading time was 5 min and 54 s with a significant difference in time for the use of devices; only flag 3 min 51 s, cattle prod 6 min 43 s and sticks 8 min 09 s. Of the carcasses observed, 772 (9.5%) had no bruises, 873 (10.7%) had one bruise, 1312 (16.1%) two, 1231 (15.1%) three and 3944 (48.5%) had four or more bruises. Prevalence of bruises were highest on the *Tuber-coxea* (hip) (29.3%) following forequarter (22.4%), *Tuber-ischiadicum* (rear) (17.3%), ribs/flank (14.1%), rump/round (10.1%) and loin (6.8%). Bruises were 68.7% grade 1 and 31.3 % grade 2; there were no grade 3, the deepest ones, observed. It appeared that animal welfare training of truck drivers worked out well and the use of flags increased compared to a previous study in 2008.

## 1. Introduction

In Uruguay, beef production is an important part of the economy. In 2016, beef was the main export product, and Uruguay is the 6th largest beef exporter in the world, with a US $1000 million income [1]. Worldwide product quality is crucial for the export position, and animal welfare is part of this condition [2]. In the extensive production system used in Uruguay, which is generally welfare friendly, one of the most important stressors for cattle is the transport to the slaughterhouse [3]. These animals are not used to being handled by humans and therefore handling animals on farm, loading and unloading from vehicles, transportation, occasionally passing through livestock markets, and lairage can all affect their welfare [4]. Furthermore, the fasting during transport and stunning at the slaughter affect welfare as well. Reducing physical and emotional stress during transport and associated events can improve both carcass quality and animal welfare [5].

In 1986 welfare was defined by Broom [6] as follows: “The welfare of an individual is its state as regards its attempts to cope with its environment. This includes both the extent of failure to cope and the ease or difficulty in coping”. Until the moment of transportation, most beef cattle in Uruguay is reared extensively on pasture or silvopastoral systems. In this situation an animal can adapt to conditions. The deprivation of food and water, fear, arousal, mixing of groups, physical exertion and injuries are all factors that contribute to stress during transport [7]. The inability to adapt to living conditions during transport makes transport a severe impairment in welfare [8,9]. What events contribute most to the loss of welfare, and what measures are most effective to limit this degradation, are not determined yet.

There are two ways carcass bruising influences the value of marketed beef. First, severe bruises are removed from the carcass, resulting in a loss in weight per carcass [10]. Second, the increasing public concern of beef production, the way animals are slaughtered, and the degradation of welfare documented by bruises, can influence the market, and affect total demand for beef. [11]. Economic losses to bruises vary. A study from New-Zealand from 1977 reported 5.58 kg of bruised meat removed from each carcass [12]. The average loss per carcass in Uruguay was found to be 1602 ± 212 g with a minimum of 50 g and a maximum of 4900 g [10]. Numerous studies have been conducted worldwide to assess the welfare of cattle during transport [13,14,15,16]. Physiological parameters, carcass quality and behavioral measures are used for the assessment. Most studies use carcass quality since this is the least invasive method. The physiological responses to handling and transport of different livestock production animals was also extensively documented [9]. Bruising, as an event during transport, increases cortisol, packed cell volume, lactate dehydrogenase isoenzyme and hearth rate, as well as behavior [13,14,15,16,17].

Studies of events that caused bruising were made through direct observations and video analysis of 52 selected cows [18]. They found that 46.1% of the bruises were inflicted during animal-facility interactions, 26.9% from human-animal interactions, and another 26.9% from animal-animal interactions. The potential bruising events occurred mostly during the lairage time (91.2%), and this was confirmed by other authors [19], who added load density and stops during transportation of the cattle as risk factors for bruises. Only bruises on the back were inflicted in the stunning box and most bruises on the pin area were inflicted during loading at the farm [19]. For several years it has been assumed that the welfare of cattle depends greatly on the attitudes and training of stockpersons and on the availability of appropriate facilities [16]. Also, extreme temperatures, below −15 °C or above 20 °C, can influence cattle during transport as well as the experience of the truck driver [13].

It has been reported that factors that were associated with a high number of bruises were bad maintenance of the truck, presence of guillotine doors, journey duration longer than 5 h, bad quality of roads, devices to move animals and the presence of horned animals [3]. The objective of the present study was to evaluate whether certain events during loading, transporting, and unloading cattle have an influence on the amount, location, and degree of bruises on the carcass.

## 2. Materials and Methods

Over a period of 3 weeks all trucks arriving at one of the major slaughterhouses of Uruguay have been assessed on various parameters in three main subjects: loading, the journey, and unloading. The following day, all animals were assessed on the number, location, and severity of bruises on the carcass according to the method developed previously by Huertas et al. [3]. All assessments were done by four trained observers. To standardize observations in the slaughterhouse, a two-week trial period was performed, in which the inter-observer reliability was optimized. The daily routine of the slaughterhouse was as follows: Most cattle transports arrived between 5 pm and 2 am. After arrival and administration, the truck driver would unload the cattle on a ramp. Animals were given a group number when leaving the truck. This number was related to the farm of origin. Some groups were on multiple trucks and on some trucks there was more than one group present. All groups of animals stayed together and there was no mixing between groups. From this point on the lairage period started in which animals where moved to different pens closer to the stunning box. Slaughter started at 6 a.m. with an all-in-all-out system. Information about the loading and the journey was collected with a survey from the truck drivers.

### 2.1. Truck, Driver and Loading

The loading of the cattle was assessed from a survey filled in by the truck drivers, asking for: Time the loading took in minutes; the course of loading specified between ‘good’, ‘regular’ and ‘bad’; the use of the following devices: flags, electric cattle prods and/or sticks. Furthermore, the questionnaire was about the number of years of experience with transporting beef cattle and if an animal welfare training course was attended.

### 2.2. Unloading

After arrival of the cattle at the slaughterhouse, the course of unloading was assessed. The unloading was scored on way of parking; unloading time in seconds; the behavior of animals on the truck and the behavior when animals were leaving the truck; the use of devices to force animals to move i.e., flags, electric cattle prods and sticks; the manner in which these devices are used i.e., gentle (soft, with a soft touch, just to persuade), intense (stronger than before, but without damaging) or rough (rude, with excessive force, causing damage); the number of people that were involved unloading the cattle and the use of hard shouts and hard sounds. Parking was considered correct if there was <5 cm difference between the trucks and the ramps edge in distance and/or sideward deviation. Behavior was scored and considered nervous if there were one or more animals vocalizing repeatedly, running, and/or jumping. The use of devices to force animals to move i.e., flags, electric cattle prods, and sticks; the way these devices are used i.e., gentle if these were used only as a touch and less than five times, intense if they were used between 5 and 10 times and rough if the devices were used more than ten times and in a hard way per load. The use of sticks usually meant handling a flag as a stick by turning it around or a stick. Shouts were considered used if a person handling the cattle used their voice loudly to urge on cattle. Sounds were assessed similarly, and these were mostly created by hitting devices against the truck.

### 2.3. Carcass Scoring

At the slaughter line the carcasses were assessed on location and degree of bruises, according to a method developed previously by some authors [3]. Only bruises and lesions with signs of live tissue damage were included. There were three types of lesions distinguished in this study: Grade 1 only superficial tissue is involved, with a diameter less than 10 cm. Grade 2 bruises involved damage to underlying muscle tissue and contained all lesions bigger than 10 cm in diameter. Grade 3 very deep (affecting muscle and even bone), partial or total condemnation of the carcass. Bruises localization: Region 1 is the forequarter, region 2 the ribs and flank, region 3 rump and round, region 4 is the loin. A special category was made for the region of the *Tuber coxae* (hip) and *Tuber-ischiadicum* (rear) i.e., region 5 and 6 respectively. This category was introduced because of the frequent occurrence of lesions. If a bruise or lesion occurred in more than one area the bruise was only recorded in the area where the largest part of that bruise was located.

### 2.4. Data Analysis

All results were analyzed using IBM SPSS (version 20). For descriptive analyses, different methods have been used. Most continuous variables are described by mean, maximum and minimum. For discrete variables a proportion was determined.

## 3. Results

In total 242 trucks were used in the analysis. They carried 8132 animals to the slaughterhouse. These were European breeds of an average weight of 450 kg.

### 3.1. Loading

Average loading time was 26 min and 21 s (from 00:06:00 to 05:00:00) where cattle was loaded from different farms. Course of loading was assessed by the truck drivers, as being good in 81.9%, neutral in 15.7% and bad in 2.4% of cases. According to them, in 10.6% of the loading events no devices (electric cattle prods, sticks or flags) were used, one device in 44.4%, two in 43.2%, three in 10.0%, and four in 2.9% of the events. The devices most used by the drivers to load cattle on the truck were flags (81.7%), sticks (9.1%), electric cattle prods (31.5%) and shouts (49.8%). In 39.3% of the cases only a flag was used as see in Table 1.

**Figure 1:**
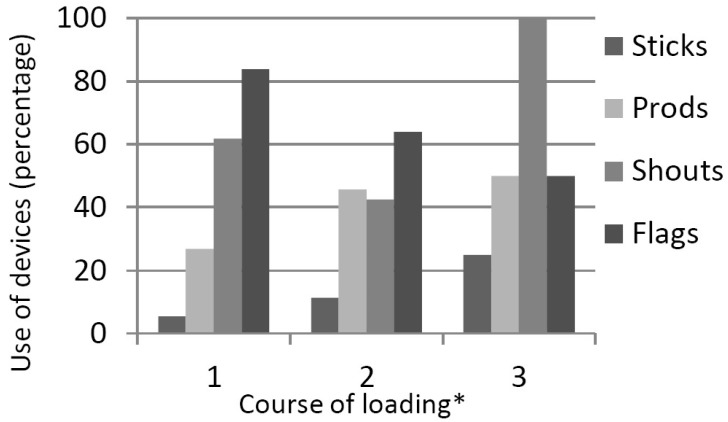
The use of devices in different courses of loading as interpreted by truck driver. *: Interpretation course of loading by truck driver (1 = good, 2 = neutral, 3 = bad).

Figure 1 shows the trend from which can be concluded that the use of devices such as electric cattle prods and sticks or the use of hard shouts were associated with a neutral or bad course of loading. The use of flags is negatively correlated with the course of loading by the drivers (r = −0.218 *p* = 0.007) and the use of prods (r = 0.189 *p* = 0.022) and shouts (r = 0.197 *p* = 0.022) are positively correlated with the course of loading. The course of loading is expressed as 1 = good/smoothly, without problems; 2 = neutral; 3 = bad/long time needed with many problems.

### 3.2. Truck Driver

Years of experience transporting cattle was on average 17.4 years with a maximum of 40 years of experience. An animal welfare course was followed by 79.5% of the truck drivers.

### 3.3. Unloading

Truck parking at the ramp and cattle unloading were observed by the researchers. It was assessed as correctly in 93.7% of all cases. The behavior of the animals on the truck was calm in 69.0% of all cases, when leaving the truck, 80.3% of all cases behaved calm. There was a correlation between the behavior on the truck and when leaving the truck, (r = 0.294 *p* < 0.000). The average unloading time was 5 min and 54 s (±03:54) with a minimum of 01:09 and a maximum of 30:21. In 79.6% of unloading events sticks and/or electric cattle prods were used to force animals off the truck as see in Table 2. Hard shouts were used in 59.5% of all cases and hard sounds in 19.4% of all cases. The average number of people interfering with the unloading of cattle was one in 70.7%, two in 26% and three or more in 3.3% of all unloading events.

In Table 3 the average number of bruises per animal is presented in relation to the various ways of unloading. The correlations between the various ways of unloading and number of bruises per animal were low and not significant.

### 3.4. Carcass Scoring

Of the 8132 carcasses observed, 873 (10.7%) carcasses had one bruise, 1312 (16.1%) had two bruises, 1231 (15.1%) had three bruises and 3944 (48.5%) had four or more bruises. 772 (9.5%) of the carcasses had no bruises. The average number of bruises per carcass was 3.75. The location and prevalence of bruising on a carcass is shown in Table 4. There were no grade 3 bruises in this study.

## 4. Discussion

There is a close relationship between handling ruminants pre-slaughter and the quantity and quality of the meat they produce [5]. Several initiatives, including activities focused on the impact of pre-slaughter conditions on beef cattle (facilities, equipment, and handling procedures), have been carried out in Uruguay and in the region to promote animal welfare and improve meat quality [20,21]. The results of the present study revealed that the use of a flag to move cattle had the best efficiency. The animals entered the truck quickly and calmly. In 95 (39.3%) out of 242 truckloads only a flag was used to get the animals on the truck. In 58 (24%) cases also shouting was added. This is a substantial difference to the previous findings [3] when in 75% of the cases electric cattle prods were used and in 40% shouting (no flags were used at that time). Electric prods and sticks are used less in the present study. Presumably, the drivers started to use a flag only, but added other devices when the cattle did not enter the truck quickly. These results can be explained, in part, by the diversity of dissemination, extension and training courses on animal welfare concepts and meat quality delivered to the stakeholders [22,23]. However, in the present study, more than 90% of the animals had one or more bruises and this is around 30% more than in the previous study, in which the same scoring system for carcass bruising was used [10].

The extensive way beef cattle are raised in Uruguay is such that animals do not come in contact with humans very often. Transportation is, therefore, very stressful. The use of flags to move cattle is believed to be less traumatic than the use of other devices [24]. Therefore, it was surprising to observe more bruised animals than in the previous study, where flags were not used, but electric cattle prods [3]. Although in the present study, more than 90% of the animals had one or more bruises and this is more than in the past, in which the same scoring system was used for the carcass bruises, there may have been some overvaluation of some bruises by the observers, as found by some authors [25,26]. They found “slight” inter-observer agreement for the number of bruises scored per anatomical site and “fair” for the severity grade of the bruises.

Nevertheless, no bruises of grade 3 were found, the deepest and most severe ones. This finding matches results found recently. The third quality audit of beef was conducted in Uruguay [27] by other authors in the country [28], and no bruises of this degree of severity were observed. It should be noted that in the present study, data were collected on only one slaughterhouse, whereas in the previous studies several abattoirs were visited [3,10,28]. Some researchers state that the presence of carcass bruises is affected by characteristics of the animals, the conditions of transport, the handling of the animals, and the waiting time at the slaughterhouse, more than the loading and unloading process [19,29,30]. Although the presence of horns has been reported as a problem in several countries, the results of some authors [31] suggest that there is no significant relationship between the prevalence of horns and carcass bruising, handling being a critical point. We agree that further research is necessary to evaluate the causality this problem.

When loading cattle, the interpretation of truck drivers regarding handling methods of loading was associated with the duration. A shorter loading time was considered to be good loading. The correlation between the time loading, unloading and the use of different devices is probably due to the fact that people, handling cattle, tended to start using devices if loading and unloading halted. Of all the devices used during unloading, the use of flags only had a significantly lower time unloading cattle.

Interventions at these stages have considered training animal handlers and transporters by showing them the consequences of bad handling with audiovisual material prepared on site. Research results have helped to improve animal welfare and support the development of new legislation or to make changes in the existent legislation related to animal welfare [4,20].

The way to achieve the cultural change necessary to improve animal welfare, operator safety and profitability of the sector is through training and knowledge transfer. The results show that the joint efforts of all the institutions and the active role of the World Organisation for animal health (OIE) Collaborating Center, a consortium of Chile, Mexico, and Uruguay, have been more effective, as have the continuing education programs implemented by universities [32].

Previous reports and results of this study indicate that the transport of beef cattle to the slaughterhouse is accompanied by impaired animal welfare. A tool to measure animal welfare is to look at bruises on the carcass. These bruises are an incentive to improve the transport conditions and animal welfare because this prevents economic losses. Strict guidelines on human-animal interactions and on the use of devices are important issues. From this point of view, it is good to observe that many drivers used no device (10.6%) or only a flag (39.3%) to load the cattle onto the truck.

## Figures and Tables

**Table 1:** The use of devices when loading cattle.

Device	Number	%
Sticks	1	0.4
Prods	5	2.1
Shouts	6	2.5
Flags	95	39.3
Sticks and prods	1	0.4
Sticks and shouts	4	1.7
Sticks and Flags	2	0.8
Prods and shouts	21	8.7
Prods and Flags	18	7.4
Shouts and Flags	58	24.0
Sticks and Prods and Shouts	7	2.9
Sticks and Prods and Flags	0	0.0
Prods and Shouts and Flags	17	7.0
Sticks and Prods and Shouts and Flags	7	2.9

**Table 2:** The use of devices when unloading cattle.

Device	Not Used%	Gentle%	Intense%	Rough%
Flag	44.7	15.6	29.1	7.8
Electric prod	44.4	8.3	6.3	41.0
Stick	75.7	8.6	3.3	12.5

**Table 3:** Average number of bruises per animal with the various ways of unloading.

Unloading	AVG Bruises Per Animal
Gentle	3.51
Intense	3.46
Rough	3.70
Flag	3.51
Stick	3.49
Prod	3.64
Noise	3.77
Quiet	3.19

**Table 4:** Gradation of bruises and the prevalence of the location on the carcass.

Grade	Forequarter%	Ribs/Flank%	Rump/Round%	Loin%	T. Coxae (hip)%	T. Ischiadicum (rear)%	Total%
1	12.2	7.9	8.3	5.5	20.0	14.9	68.7
2	10.2	6.3	1.9	1.3	9.3	2.4	31.3
Total	22.4	14.1	10.1	6.8	29.3	17.3	100
